# Retraction: Baicalin prevents myocardial ischemia/reperfusion injury through inhibiting ACSL4 mediated ferroptosis

**DOI:** 10.3389/fphar.2026.1794677

**Published:** 2026-02-03

**Authors:** Frontiers Editorial Office

The journal retracts the 14th of April 2021 article cited above.

Following publication, concerns were raised regarding the integrity of the data in the published Figure 1C, and the subsequent duplication 3 years later of this image (Figure1A) in another journal’s published article (Kardiologiia, 2024 October 31; 64 (10):48–56. doi: 10.18087/cardio.2024.10.n2739). The authors failed to provide the raw data or a satisfactory explanation during the investigation, which was conducted in accordance with Frontiers’ policies. As a result, the data and conclusions of the article have been deemed unreliable and the article has been retracted.

This retraction was approved by Chief Editors of Frontiers in Pharmacology and the Chief Executive Editor of Frontiers. The authors have not responded to correspondence regarding this retraction.

